# How to Sterilize Polylactic Acid Based Medical Devices?

**DOI:** 10.3390/polym13132115

**Published:** 2021-06-28

**Authors:** Sara Pérez Davila, Laura González Rodríguez, Stefano Chiussi, Julia Serra, Pío González

**Affiliations:** 1 New Materials Group, Campus Lagoas-Marcosende, CINTECX, Universidade de Vigo, 36310 Vigo, Spain; laugonzalez@uvigo.es (L.G.R.); schiussi@uvigo.es (S.C.); jserra@uvigo.es (J.S.); pglez@uvigo.es (P.G.); 2 Galicia Sur Health Research Institute (IIS Galicia Sur), SERGAS-UVIGO, 36213 Vigo, Spain

**Keywords:** medical devices, polymers, polylactic acid (PLA), 3D printing, sterilization, supercritical CO_2_, gamma irradiation, steam, ethylene oxide

## Abstract

How sterilization techniques accurately affect the properties of biopolymers continues to be an issue of discussion in the field of biomedical engineering, particularly now with the development of 3D-printed devices. One of the most widely used biopolymers in the manufacture of biomedical devices is the polylactic acid (PLA). Despite the large number of studies found in the literature on PLA devices, relatively few papers focus on the effects of sterilization treatments on its properties. It is well documented in the literature that conventional sterilization techniques, such as heat, gamma irradiation and ethylene oxide, can induced damages, alterations or toxic products release, due to the thermal and hydrolytical sensitivity of PLA. The purposes of this paper are, therefore, to review the published data on the most common techniques used to sterilize PLA medical devices and to analyse how they are affecting their physicochemical and biocompatible properties. Emerging and alternative sterilization methods for sensitive biomaterials are also presented.

## 1. Background

Sterilization is a fundamental step in the manufacturing process of any biomaterial or medical device that will be in contact with the human body, as well as in the process of reusing medical instruments [1], to avoid any complications such as infections or rejections. Sterilization is defined as the ability to eliminate or completely destroy all microbial life-forms, including viruses, bacteria and fungi, with either vegetative cells or spores [2,3]. However, because absolute sterility cannot be verified, the statistical definition of sterility used in practice is based on the Sterility Assurance Level (SAL), which for any biomedical device should be limited to a SAL of 10^−6^, meaning that maximum one viable microorganism should be found in one million sterilized samples [4]. In addition to their effectiveness, sterilization methods should not cause significant changes in the physical, chemical, mechanical and biocompatibility properties of the material, that might produce adverse responses in the body or compromise its function [3].

Despite the significant progress in the development of biomaterials in recent years, sterilization techniques have remained unchanged over time. There are three current conventional methods for sterilizing medical devices that are the most industrially used: ethylene oxide (EtO), gamma irradiation, and steam sterilization [5,6]. Unfortunately, for the sterilization of sensitive materials such as polymers, these conventional techniques have certain disadvantages which may severely alter their original properties. It is extensively proven that steam sterilization is not suitable for thermal and hydrolytic sensitive biomaterials because they do not tolerate the temperatures required during autoclaving (steam sterilization) [4]. Unlike autoclaving, gamma irradiation and ethylene oxide can be applied to thermolabile materials thanks to lower sterilization temperatures. Nevertheless, gamma irradiation can deteriorate polymers causing scission and cross-linking in polymer chains, resulting in decreased molecular weight and increased biodegradation rates [3]. Ethylene oxide is known as a polymer softener and plasticizer and is being progressively prohibited by several hospitals in the EU and USA because it is a toxic, flammable, and explosive gas that has carcinogenic and allergenic effects [6,7]. In addition, the EtO sterilized devices require long aeration processes to remove possible toxic EtO residues that can be present after the sterilization process [6]. Other sterilization techniques, such as hydrogen peroxide gas plasma (HPGP), peracetic acid, or ozone treatments are also currently being explored as alternative methods to the conventional techniques. Even though there are several technologies that can provide terminal sterilization, there is no specific technique that can be applied to all materials used for biomedical devices, because each technique has its advantages and disadvantages according to the nature and application of the material, as summarised in Table 1. Although most of the widely used methods run at low temperatures ideal for sensitive materials, they have their specific limitations, because they have been shown to alter morphology, structure, and surface properties of different polymers [5]. Therefore, research in recent years has been focussed on novel processes to sterilize polymeric materials, and to avoid the problems mentioned above, without affecting the integrity of the materials. Sterilization using supercritical carbon dioxide emerges as a green and sustainable technology, able to reach very low SALs without altering the original properties [6]. Moreover, emerging manufacturing technologies, such as 3D printing, are considered self-sterilizing by many authors, due to the high temperatures and pressures of the extrusion process [8,9,10].

As mentioned above, an efficient sterilization is one of the most important challenges for allowing the clinical application of medical devices, in particular of polylactic acid (PLA) based ones. PLA is a bioabsorbable, biodegradable and biocompatible polymer, that can be produced from sustainable sources and offers a promising alternative to traditional biomaterials and non-biodegradable polymers. Lactic acid, the main constituent of PLA, is a chiral molecule, existing as L and D isomers. That is why in this review, the term PLA include pure Poly-L-lactic acid (PLLA), pure Poly-D-lactic acid (PDLA) and Poly-D-L-lactic acid (PDLLA) [9] or PLA mixed with other materials like hydroxyapatite [10] or antibiotics [11]. PLA is degraded by enzymatic activity or by hydrolysis, forming lactic acid that is usually present in the body. In this way, inflammatory reactions are prevented, and the by-products are expelled through normal cell activity and urine [12]. It has therefore a rapid clinical translatability, is widely used and already accepted by the Food and Drug Administration (FDA) in almost all medical specialties: orthopaedic applications (scaffolds, bioabsorbable screws, guided bone regeneration); cardiac applications (stents); dentistry; plastic surgery (sutures, dermal fillers, etc.); and systems for drug release [13,14].

Recently, the interest in PLA based medical devices has dramatically risen, since PLA is a suitable material used in emergent technologies such as 3D printing. Its properties and its low glass transition temperature (55–65 °C) makes it deformable under high temperatures (190–220 °C) converting it in one of the most used filaments in this technology. The use of 3D printing has been well accepted in the biomedical field and it is already possible to see 3D printers in many hospitals. One of the most widely used techniques is the fused deposition modelling (FDM) technique, that allows the rapid manufacture of customised structures with complex geometries and excellent reproducibility, allowing a personalised medicine. It is already applied in numerous domains both in research and in surgical practices, such as patient-specific implants, surgical guides (cranial and maxillofacial surgery) and surgical tools, improving medical outcomes and decreasing radiation exposure for patients [15,16,17].

Moreover, the effectiveness and usefulness of these manufacturing technologies has been demonstrated not only in remote medicine but also in emergencies. Due to the coronavirus disease in 2019 (COVID-19), 3D printing has had a major impact on our society, playing a key role in the provision of personal protective equipment (PPE). Where volunteers and professionals manufactured half-face mask, safety goggles, and a face-protecting shield, among others, to meet the high demand. Although these products can be disinfected according to traditional protocols, it is very important that parts to be used in surgical operations or in contact with tissues must be properly sterilized. In 3D printing, the dimensional accuracy after sterilization (e.g., critical in fan connectors), the chemical or thermal resistance of materials where only autoclaves are available, are examples of key parameters [18,19].

It is remarkable that despite the large number of studies that propose PLA in multiple applications, only few of them systematically investigate how different sterilization treatments can affect the properties of PLA based materials. This paper reviews the most relevant studies over the last 30 years which cover the different methods used to sterilize polylactic acid polymer (PLA) based materials (Table 2). Comparisons of these methods, their sterilization mechanism and post-sterilization effects on physicochemical and biological properties will be discussed.

## 2. Heat-Based Sterilization Methods

Fundamentals of sterilization by heat are based on the destruction of essential metabolic and structural replication components of microorganism due to direct heating and oxidation effects, with two methods: steam and dry heat sterilization, using saturated steam between 120 °C and 130 °C for 20 min (autoclave) or hot air at 160 °C for 2 h, respectively. The advantage of these methods is that they are effective, fast, simple, do not leave any residues and have excellent penetration [8]. These methods can therefore be considered the gold standard of sterilizations since any research laboratory or hospital has equipment to heat sterilize their mostly metal-based medical instruments. However, it is well established in the literature that it cannot be applied to heat sensitive polymers. Most biodegradable polymers used in biomedical applications, such as PLA and PLGA, do not survive a standard autoclave cycle, since this combination of severe heat and moisture results in an extensive thermal and hydrolytic degradation. Moreover, exceeding glass transition and melting temperatures may result in a mechanical deformation of the devices [3,4,20].

Gogolewski and Mainil-Varlet tried to lower the temperature of the dry heat process by applying vacuum or inert gas atmosphere, to avoid, unsuccessfully, the decreases in PLA molecular weight and changes in mechanical properties [21]. On the other hand, as the damage caused by steam sterilization in polymers was already well-known, in 1991, Rozema et al. studied the effects of different steam-sterilization programs on the properties of poly (L-lactide). However, in the different designed autoclave programmes the molecular weight always decreased after sterilization, being the one producing less alterations a programme using short time (60 s) at high temperature (129 °C) [22]. This special autoclave cycle was also used by Cordewener and colleagues, corroborating that extracts from poly (96 L/4D-lactide) blocks show no cytotoxicity after sterilization [23]. In the same way, the researchers Filippova and Ivanova, showed the possibility of autoclaving PLA films for corneal implants. Although the autoclaving process increased the hydrophilicity and the internal surface roughness by up to five times [24], no inflammatory reactions or increased eye pressure were observed [25] in animal models.

**Figure 1:**
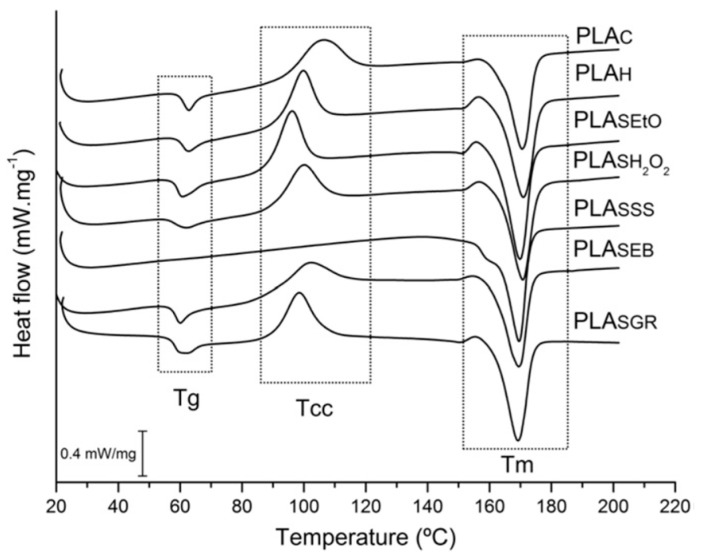
PLA DSC thermogram before and after the hygienization process and the five sterilization processes. (PLAC) control, (PLAH) hygienized, (PLASEtO) sterilized with ethylene oxide, (PLASH2O2) sterilized with hydrogen peroxide plasma, (PLASSS) sterilized with saturated steam, (PLASEB) sterilized with electron bean radiation, (PLASGR) sterilized with gamma radiation. Reprinted from M. Savaris, V. dos Santos, R.N. Brandalise, *Mater. Sci. Eng. C* 2016, 69, 661–667 [26]. Copyright 2019, with permission from Elsevier.

Other studies, on the contrary, do not recommend using this method. Savaris and collaborators [26], for example, analysed the modifications in the properties of commercial poly (lactic acid) films after exposure to five different sterilizations techniques. Saturated steam sterilization caused the deepest changes and even damaged the PLA. Optical microscopy micrographs and Fourier-transform infrared (FTIR) spectroscopy analysis indicated surface modifications and changes in chemical structure, respectively. As they describe, the possible start of fusion, resulting from the high temperature and pressure of the process, caused holes in the surface and of peaks in the FTIR, corresponding to signs of hydrolytic degradation which simultaneously cause a whitening of the sample. With hydrolytic degradation, an increase in crystallinity is typical, in this case from 11.9% to 60.5% with respect to the control sample. The disappearance of a measurable glass transition (Tg) and a cold crystallisation (Tcc) temperature in the Differential Scanning Calorimetric (DSC) thermograms, shown in Figure 1, also confirm the crystallization of the original amorphous PLA during sterilization with saturated steam (PLASSS), as the crystallisation temperature was exceeded (over 106 °C) [26]. Similar to this study, Zhao et al. investigated the effect of several heat sterilization methods in commercial biodegradable PLA for single-use, disposable medical devices. As previously commented, all changes observed are attributed to the fact that the material already crystallized during sterilization. Among these changes are: the decrease in strength, increase in rigidity as well as the opacity and shrinkage of the samples, since an amorphous sample is usually transparent but gets opaque after sterilization. In the DSC curves appears a weak Tg peak and a strong melting peak at Tm, with the disappearance of the cold crystallization peak. The results of Wide Angle X-ray Diffraction (WAXD) and FTIR, confirm that autoclaving leads to the crystallization of PLA, with the appearance of an absorption band corresponding to the characteristics of crystalline alpha-phase PLA (922 cm^−1^) and a decrease in the band of the amorphous fraction (956 cm^−1^) [27]. Similar effects were reported by Rainer et al., with the autoclave cycle and dry heat sterilization, inducing an increase in crystallinity of PLLA electrospun scaffolds [28].

There are also specific studies on the effect of autoclave sterilization on PLA models, scaffolds or devices made with 3D printing. Aguado-Maestro confirms that autoclaving completely deformed their PLA cylinders in size or shape, not being an exact replica of the original STL file created by computer [15]. Boursier et al. also studied the effect of the autoclave sterilization on 3D-printed bone models by measuring changes in size. Contrary to Aguado, their results showed that the model printed before and after sterilization are the same, with strong or perfect accuracy. These results differ from those of the previous authors, as they validate the autoclave as a sterilization method without observing any changes in the bone models after the process. However, they indicate that the effects of autoclaving could be more harmful on a surgical guide or some larger model since in their study they focused on the cat tibia, which is a relatively small bone. In addition, an exhaustive study of the physicochemical changes was not carried out [29].

Ferrás-Tarragó et al. indicated that the previous studies do not assess the biosafety or mechanical consequences of post-printing strength and printing parameters, which are very important for 3D-printed surgical tools and devices [30]. In fact, they proposed a protocol for defining the sterilization by autoclave of PLA fracture biomodels during preoperative planning. The parts were sterilized at 134 °C and subjected to a 3D scanner before and after sterilization to see changes in area and volume, as well as deformations. They indicate that, if their protocol is strictly followed and the filler percentage is less than 25%, any biomodel 3D-printed with PLA would have an autoclave sterilization success rate of almost 100%, without significantly altering the morphology of the biomodels. [31].

Frizziero and co-workers were able to produce and sterilize by autoclaving 3D-printed custom cutting guides (CCGs) for paediatric orthopaedic surgery maintaining their mechanical properties and the design geometry of the original design. Although they do not specify the tests performed in their study, the fact that the autoclaving process did not affect the guide was because they used a high-temperature PLA fibre (HTPLA) with thermomechanical properties that can withstand common steam sterilization without bending or losing the original geometry [32].

## 3. Chemical and Plasma-Based Sterilization Methods

### 3.1. Ethylene Oxide

Ethylene oxide (EtO) is commonly used to sterilize a wide range of medical and clinical products, since the 1950s. Due to its low temperature and its good penetration properties, EtO is one of the most suitable processes for the majority of heat- and/or moisture-sensitive medical products. In an EtO chamber, the product is exposed to a validated combination of humidity (40–80%), EtO gas (450–1200 mg/L), temperature (37–63 °C), and time (1–6 h). The mechanism of microbial kill is alkylation of the cellular molecules that may contain amino, carboxyl, thiol, and amide groups, resulting in permanent suppression of cell metabolism and division. Although EtO can be used safely in accordance with the appropriate regulations, its use has been limited due to its hazardousness to both humans and the environment as it is a flammable gas with carcinogenic and mutagenic potential. EtO leads to changes in the polymer structures, causes molecular weight loss and the residual toxicity that may remain in the material after sterilization is also a concern [3,4,8,20].

As detailed in their study, Hooper and colleagues showed that it is possible to sterilize PLLA with EtO without affecting the molecular weight, surface composition, mechanical properties, or degradation rates [33]. The same was suggested by Savaris and collaborators where the changes of the morphological, physical, chemical, and thermal properties of commercial poly (lactic acid) films, were not significant [26].

**Figure 2:**
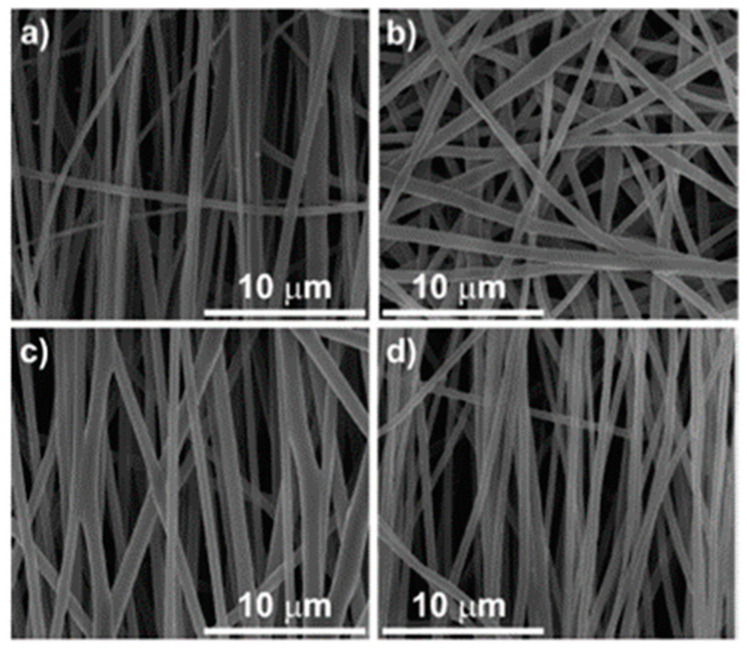
Morphology of aligned PLA fibres: (**a**) non-sterilized, (**b**) sterilized by ethylene oxide, (**c**) sterilized by UV radiation, (**d**) sterilized by gamma-rays radiation. Reprinted with permission from T.A.M. Valente, D.M. Silva, P.S. Gomes et al., *ACS Appl. Mater. Interfaces* 2016, 8, 3241–3249 [35]. Copyright 2016 American Chemical Society.

However, other authors discarded this method because of the following modifications found: Weir and co-workers observed a slight decrease in molecular weight and an increase in crystallisation of PLLA pellets after sterilization by EtO, that could alter the rate of degradation [34]. Zhao et al. also studied the effect of several methods of sterilization in commercial biodegradable PLA for single-use, disposable medical devices. They studied the modifications in transparency, yellow index, dimensional stability, and mechanical properties. They ruled out this method as well as the autoclave for PLA sterilization. Although no further significant changes were found, a contraction of the same level as using the autoclave was detected (around 0.8%) that can be attributed to the relaxation of molecular chains close to the glass transition of the polymer [27]. Valente et al., analysed the effect of three sterilization methods on aligned PLA fibre membranes and also discarded this method [35]. As shown in Figure 2, sterilization by ethylene oxide causes a total loss of orientation of the fibres and a change in shape from cylindrical to ribbon-like structures, combined with a 28% higher polymer crystallinity, that could have a negative effect on cell adhesion and proliferation. In contrast to these studies, and focussed on 3D manufacturing, Aguado-Maestro recommends EtO to sterilize non-solid surgical guides and biomodels made with PLA because, compared with steam heat and gas plasma, the EtO treatment was the only one without any Colony Forming Unit (CFU) or visual deformations [15]. Nevertheless, a more detailed analysis of physical-chemical properties and toxicity is required.

In addition to the possible damage described above, the major disadvantage of this method is that residues remain inside the device and subsequently react with the proteins in the tissues, when implanted. It has been shown that these residues can be retained for 1–3 months and cause inhibition of embryonic development in bovine models, even after prolonged aeration and repeated washings [36]. However, regarding biocompatibility studies with PLA, no cytotoxic effects were found with this method. Cordewener and colleagues analysed the cytotoxicity of PLA blocks extracts at different degradation times, after sterilization by gamma radiation, EtO and autoclave. The observed cytotoxicity was not due to the sterilizations itself, but to changes in the pH and osmolarity of the products accumulated in the different states of PLA degradation [23]. Identical to this, Savaris et al. showed that the PLA films treated with EtO did not exhibit cytotoxicity or chemical and thermal changes. They only described a slight inflammation of the tissue with the presence of fibrosis at 21 days, as expected after the implantation of a foreign body [37].

Other types of chemical sterilizers for PLA have also been reported in the literature. According to Rankin et al., PLA surgical retractors manufactured by FDM were sterilized by glutaraldehyde, a chemical sterilant that is applied at room temperature using a 2.4% glutaraldehyde solution with a pH of 7.5 for 20 min at 25 °C, in accordance with CDC guidelines for critical medical devices. They found traces of bacterial nucleic acids after this sterilization, that were attributed to the high sensitivity of the test (detecting traces belonging to dead bacteria). They also demonstrated that the sterile retractors are strong enough for the demands of the operating room, according to the mechanical tests carried out [38]. Rainer et al. sterilized PLLA scaffolds by soaking in pure ethanol (3 immersions of 3 min with a final rinsing in mQ water). Despite ethanol soaking treatment being commonly considered a good sterilization method for polymers, severe limitations in terms of bacterial contamination have been detected, and the sterility was not confirmed due to the presence of microorganisms after sterilization [28]. This may be reason why some authors classify it as a method of disinfection rather than sterilization [20].

Another chemical sterilizer is the hydrogen peroxide (H_2_O_2_), which is an oxidizing agent that can be used in two ways to sterilize medical devices: vaporized hydrogen peroxide and low-temperature hydrogen peroxide gas plasma (HPGP), a mixture of chemical and gas plasma sterilization that will be discussed later. Sosnowski and Morrison used H_2_O_2_ steam as a method of sterilizing printed PLA parts with 1 wt% gentamicin antibiotic for biomedical use. This sterilization method exposed the samples for 28 min to a temperature of approximately 50 °C, causing changes in mass and dimensions, as well as a significant worsening of mechanical properties. In addition, a brightening of the samples was observed, associated with the known bleaching effect of H_2_O_2_ and the solubility of gentamicin in water [11].

### 3.2. Hydrogen Peroxide Gas Plasma (HPGP)

This method is a synergism between the hydrogen peroxide chemical sterilant and a low-temperature gas plasma, used in the STERRAD^®^ plasma sterilization system. Since its first use in the medical field in the early 1990s, HPGP has been considered as a promising method if the temperature does not exceed 50 **°**C and the humidity is minimal; thus, it can be applied in most medical devices that are not compatible with heat and other chemicals. Furthermore, unlike ethylene oxide, it does not leave toxic residues, is not hazardous, but easy to store and does not require aeration time. The mechanism of this process is the combination of a UV irradiation generated from excited gas molecules (with radio frequency or microwaves) and the resulting free oxygen radicals, causing lesions of DNA strands in microorganisms. This method is used predominantly in hospitals as a surface sterilization technique for implantable devices and polymers and less in medical devices manufacturing [3,4,36,39].

In the reviewed literature, studying hydrogen peroxide plasma gas (HPGP) as a sterilization method, four of the studies used different models of STERRAD^®^ (Johnson and Johnson) sterilizers [26,28,36,39] and one used a Zerome 130 sterilization chamber (Tuoren Group, China) [27]. In all studies, the temperature to sterilize the different materials never exceeded 5 °C and a single sterilization cycle was used, where the temperature varied between 28 °C and 50 °C. Even though one cycle is sufficient for sterilization, Peniston et al. decided to apply two cycles to study in detail the changes that could occur in sterilized pure PLLA. They used the HPGP sterilization, taking the sterilization by EtO as a control, where the characterization of the thermal, mechanical and morphological properties indicated that there were no major changes with respect to the EtO sterilization. However, a significant reduction in the surface energy was also observed, suggesting the formation of polar groups, although chemical analysis by ATR-FTIR did not indicate significant changes. In addition, intermediate levels of absorbed hydrogen peroxide residuals were detected in the polymer [36]. The study of Rainer et al. reveals that HPGP is an effective sterilization in PLLA electrospun scaffolds with a standard sterilization cycle at 45 °C for 45 min (STERRAD), without affecting their chemical and morphological features [28]. Savaris and collaborators analysed the modifications in the morphological, physical, chemical, and thermal properties of commercial PLA films after exposure to five different sterilizations. Regarding HPGP sterilization, no changes in surface structure or morphology were observed, but it caused variations in crystallinity, colour, and contact angle. A reduced Tg value was observed when compared with the control (0.8 degrees lower). This reduction implies increased flexibility of the polymer chain, which consequently entails higher segment mobility and the possibility of easier crystallization. However, these changes were not significant, making this process valid to sterilize PLA [26]. Zhao et al. also studied the effect of several methods of sterilization in commercial biodegradable PLA, such as the modifications in transparency, yellow index, dimensional stability, and mechanical properties, without finding any significant change through HPGP in PLA sterilization. However, this method is not recommended for mixtures of PLA and poly (butylene adipate-co-terephthalate) (PBAT), another biodegradable polyester, due to a colour change (yellowing phenomenon). Although no significant changes in chemical structures were detected by FTIR, the yellowing can be produced by oxidation of conjugated aromatic systems induced by the oxidation by this method [27].

With respect to 3D manufacturing, an evaluation of the morphological deformation of 3D-printed genioplasty guides in PLA after HPGP sterilization was carried out by Oth and collaborators. They found statistically significant differences but in the sub-millimetre range (less than 0.2 mm) which makes them acceptable for surgical use avoiding the deformation caused by an autoclave [39]. Aguado-Maestro did not recommend gas plasma (without specifying which gas they used) for sterilising non-solid surgical guides and biomodels made of PLA as they detected Colony Forming Units (CFU), probably due to the lower penetrability of this technique compared to EtO. Gas Plasma could be used in solid models or in devices printed under sterile atmospheric conditions, such as under a laminar flow hood [15].

Stepczynsja evaluated the effect of low temperature plasma used on different microorganisms to sterilize PLA. The study confirms that this method causes up to 100% mortality of microorganisms. As described, this method is a surface modification method [40], and it would be interesting to study this process further and whether it is required to completely sterilize the samples. A further study has been found using plasma to sterilize high-temperature and pressure-sensitive agents through oxygen. As documented by Eisenbrey et al., the O_2_ plasma sterilization, as long as the parameters are chosen with care, can sterilize PLA ultrasound contrast agents without sacrificing ultrasonic properties [41].

## 4. Irradiation-Based Sterilization Methods

### 4.1. Gamma Radiation

Irradiation methods offer low temperatures, short processing times, and comparatively lower cost of operation, making irradiation techniques promising for medical products. Gamma irradiation is the most employed form of ionizing irradiation sterilization with high penetration ability. Gamma rays are high-energy photons that cause damage to DNA and prevent DNA replication, leading to inactivation of microorganisms. Unlike ethylene oxide, it is non-toxic and does not require long aeration times. However, gamma radiation can cause degradation of polymers by cross-linking, chain scission or a combination of both. Chain scission occurs in weak polymer bonds as a random rupturing, resulting in the reduction of the molecular weight and in the formation of shorter chains, that can further undergo degradation. While crosslinking results in the formation of large three-dimensional networks, leading to brittleness, discoloration, cracking, and degradation of the polymer [3,4,8].

In all papers reporting the sterilization by gamma radiation, a ⁶⁰Co radiation source was used at a dose level of 25 kGy as recommended by the International Organization for Standardization (ISO) for health care products [23,26,35,42,43,44,45]. It has been known since the late 1990s that gamma radiation produces significant changes in the properties of PLA. Hooper and collaborators showed in their study a correlation between molecular weight loss and increased dose of gamma radiation, as well as changes in mechanical properties and in vitro degradation rates [33]. In addition, Türker and colleagues sterilized samples of PLA and PLAG at four different dose levels (5,10,25,50 kGy) where sterility was achieved from 10 kGy. They detected a decrease in pore size in PLA after irradiation and an increase in surface roughness that is related to chain scission due to radical formation [46]. These data coincide with the study by Suljovrujić and co-workers where they saw that the pore size of hydroxyapatite/PLLA composites are further compromised by a higher dose of radiation (50 kGy), in addition to many other dose dependent changes, at almost all structural levels [10]. Despite the changes reported in these two articles, they validated this sterilization method since the effects of the radiation are small up to the amount of irradiation needed for sterilization (25 kGy). Türker and co-workers, observed only slight differences in further properties of PLA and PLAG, being, the latter, selected as the most gamma stable membrane according to the FTIR, DSC, TGA, and scanning electron microscope (SEM) results. The pore size decreased in PLA due to the radiation sterilization and the membrane surfaces became rougher probably due to significant scission of polymeric chains [46]. Torres-Giner et al., who used this method to sterilize PLA base ultrathin fibres, did not report changes in morphology, composition and thermal properties or cell growth viability [45]. Soriano studied the influence of gamma sterilization on PLA and PLLA release rods, and although gamma irradiation caused a reduction in the molecular weight of both polymers, the loading efficiency of fluconazole did not change after sterilization, validating the method for this particular application [44]. Accordingly, the changes found by Savaris and collaborators were not significant (variations in crystallinity, colour, and contact angle), also validating the process to sterilize PLA films [26]. Likewise, one of the methods proposed by Valente and co-workers in their study is the sterilization of on aligned PLA electrospun fibre membranes using gamma radiation, where morphology and alignment of the fibres were not affected (Figure 2), nor were the mechanical and thermal properties. This method allowed the adhesion and proliferation of osteoblastic cells, observing a close relationship in the growth and orientation of the cells when the fibres are aligned [35].

It can be deduced from the literature that gamma radiation has no effect on the biocompatibility of PLA. Cordewener and colleagues analysed the cytotoxicity of extracts at different degradation times of PLA blocks, after sterilization by gamma radiation (together with autoclave and EtO as shown in the previous sections). Again, they associated the observed cytotoxicity with changes in pH and osmolarity of the accumulated products at the different degradation states, not with the sterilization process. However, in this study, they only focused on cytotoxicity and not on physicochemical changes that could be caused by the different tested sterilizations methods, since no unsterilized sample was placed as a control [23]. Dorati and collaborators designed 3D scaffolds and 2D films with a PLA graft copolymer for tissue engineering. Although they performed a physico-chemical characterisation of the material, they only studied sterilization by gamma radiation to see changes in cytotoxicity. They observed an increase in cell viability with respect to untreated films, since the material is biocompatible and non-toxic after radiation [42]. Following this, the gamma irradiation of PLA coated with plasma polymerized Allylamine fibre meshes (70:30) did not change the cellular spreading [47], and the study by Gremáre et al. resulted in an absence of cytotoxicity and good biocompatibility including a colonisation of bone cells in the PLA scaffolds. Nevertheless, they did not study possible changes in PLA properties after gamma radiation [43].

### 4.2. Electron Beam Radiation

Electron beam (E-beam) is also an ionizing radiation, but with lower penetration than gamma and higher required dosage rates. The energy needed for sterilization ranges from 5 MeV to 10 MeV and doses range 10 kGy to 60 kGy. E-beam sterilization uses high-energy beams from accelerators to irradiate biomaterials or medical devices in their final packages and inactivates micro-organisms by the same mechanisms as gamma irradiation [3,4]. It is important to note that for this sterilisation, the correct dose is one of the most essential factors as it is directly related to the sterility assurance level of the biomedical device and to the penetration of the electrons, as higher doses are needed for thicker products due to their lower penetration capacity [48].

As described by Loo and colleagues, PLA and PLGA films are degraded through chain scission during e-beam radiation exposure, following molecular weight decrease and radiation dose a linear relationship [49]. Zhao et al. also demonstrated that the decrease in molecular weight is induced by irradiation. They studied the effect of dose ranging frome 25 up to 100 kGy and found a severe reduction of glass transition and melting temperatures, as well as a severe reduction in the mechanical properties of commercial PLA, when subjected to higher doses of electron beam irradiation [48]. In other articles focussed on sterilization by e-beam radiation, the samples were irradiated with the aid of one or two linear electron accelerators with 10 MeV energy and with radiation doses of 25 kGy, according to ISO 11137-2:2006 [27,37]. Savaris et al. and Zhao et al. concluded that E-beams can be applied for the sterilization of PLA, despite that in the latter, they found a decrease in molecular weight indicating sample degradation [27].

Benyathiar et al. determined the effects of gamma and electron beam irradiation of PLA films with different intensity dose levels of ionizing radiation (1, 5, 10 and 30 kGy) used commercial sterilization processes as well as the stability of the samples at 3, 6, and 9 months. They found changes indicating degradation of PLA by irradiation, such as a decrease in molecular weight. Other changes in mechanical, thermal, and permeability properties were also reported [50].

### 4.3. Ultraviolet Radiation

The ultraviolet radiation results in excitation of electrons and of photolytic reactions, causing the inactivation of microorganisms. It presents a very low penetration power compared to the previous radiation techniques, limiting its use to sterilize surfaces. Although this method is classified by some authors [20] as a disinfection method, some papers have explored this method for use in sterilization. In three of them, the materials were sterilised under a UV lamp of 30 W integrated in a laminar air-flow hood, with a distance of 60 cm [21,51] or 30 cm [35] between the materials and the lamp. Fischbach and collaborators studied how the UV radiation affects the diblock copolymer of Me.PEG-PLA, at different radiation times (0, 2, 5, 10, and 24 h). They observed that after the 2 h of irradiation required for sterilization, neither the physico-chemical properties nor the cell adhesion of 3T3-L1 pre-adipocytes were altered, compared to the control. However, once the radiation time was further increased, time-dependent changes in the copolymer surface occurred, affecting cell/protein-polymer interactions [51]. In accordance with this, Janorkar and co-workers observed a decrease in the molecular weight of PLA films as the time of exposure to such radiation increases up to 40 h, without changing the hydrophility of PLA [52]. Finally, Valente and co-workers also proposed this method to sterilize aligned PLA fibre membranes for 60 min. As with gamma radiation, the morphology and alignment of the fibres were not affected (Figure 2), nor were the mechanical and thermal properties. Only a slight increase in wettability was reported and like gamma radiation, the membranes had a good cellular response [35]. The study of Rainer et al. reveals that UV irradiation is an effective sterilization in PLLA electrospun scaffolds with 20 W lamp for 20 min at a distance of 20 cm [28], without affecting their chemical and morphological features. This shows the great importance to fine control the exposure time of UV radiation, to guarantee the sterilization without compromising the properties of the polymer using short times like 1 and 2 h.

## 5. New Approaches and Alternatives

### 5.1. Supercritical CO_2_ Sterilization Method

Today, biomedical devices tend to be more sophisticated and complex in material composition and structure, surface morphology and porosity, as well as their design. The need to find new and effective sterilization alternatives to avoid the damage caused by current sterilization techniques has brought the supercritical carbon dioxide (scCO_2_) technique into focus [5]. As a matter of fact, the FDA selected the company NovaSterilis for the development and regulatory of this technique as a new sterilization method and technology in the innovation challenge in 2019.

This method uses carbon dioxide in a supercritical state, at a critical point in pressure (7.39 MPa) and temperature (31.05 °C), to obtain low viscosity and zero surface tension, thus promote the penetration into most complex and porous structures. ScCO_2_ can inactivate a wide variety of microorganisms but is not sufficient on an industrial level of sterilization (SAL10-6) for some microbes, if used without any additives. To enhance the inactivation, sterilants that contain peracetic acid (PAA) are therefore commonly used as additive. Unlike ethylene oxide, this technique is not toxic and does not leave any residues on the materials. CO_2_ is economic, inert, non-flammable, and recyclable. Despite the precise inactivation mechanisms remaining unclear, in their review, Soares and collaborators indicated the effective terminal sterilization of this method, with a wide variety of microorganisms and even with thermally and hydrolytically sensitive biomedical polymers [3,5].

It has been demonstrated that supercritical CO_2_ sterilization of PLA achieved the complete inactivation of a wide variety of organisms [53] and even spores [54] using moderate temperatures to avoid physical and chemical damage in thermolabile and hydrolytically sensitive materials such as PLA and derivatives.

**Figure 3:**
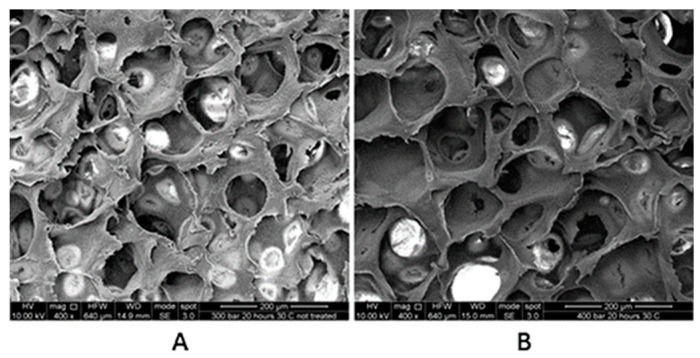
SEM images of cross sections of not treated (**A**) and CO_2_ treated (**B**) PLLA scaffolds (40 MPa, 20 h and 40 **°**C). Reprinted from S. Lanzalaco, S. Campora, V. Brucato, et al., *J. Supercrit. Fluids* 2016, 111, 83–90 [54]. Copyright 2016, with permission from Elsevier.

Dillow et al. succeeded in sterilising PLA and PLGA microspheres at 20.5 MPa and 34 °C for 30 min or 25 °C for 45 min. When comparing the sterilised samples with the control, no chemical changes were observed by FTIR, gel permeation chromatography (GPC), and DSC analyses. A decrease in molecular weight or total mass of PLGA was observed as a result of degradation in PBS, but no difference with respect to EtO and steam sterilization methods [53]. Lanzalaco and collaborators used this same method to sterilize porous Poly (L-lactic acid) scaffolds. To ensure the three-dimensional effectiveness of the sterilization, they contaminated the samples with bacteria (Escherichia coli) and spores (Streptomyces coelicolor). Unlike the bacteria, the spores required more severe sterilization conditions due to their tough structures, such as 360 min of exposure at 30 MPa, and 40 °C or 30 °C using hydrogen peroxide as an additive. The treatment did not alter the biocompatibility or the structure of the scaffolds as shown by the calorimetric and SEM analyses [54]. Figure 3 shows clearly that the sterilization did not affect the size of the pores or the morphology with respect to the control. As indicated in their study, this is a very important characteristic since it could influence cell growth during scaffold colonization.

### 5.2. 3D Printing. A Self-Sterilization Method

In the reviewed literature (Table 3), several publications can be found that demonstrate the intrinsic sterility of 3D-printed devices using fused deposition modelling manufacturing (FDM) [15,38,55]. The pieces could be manufactured and simultaneously sterilized to be used in biomedical applications, lowering costs and without damaging them by other sterilization methods [15].

Rankin and collaborators were one of the first groups to focus on 3D printing as a self-sterilization method. In their study, they manufactured PLA surgical retractors sterilized by glutaraldehyde. They realised that the filament itself, immediately collected after extrusion, revealed the absence of viable bacterial products by Polymerase Chain Reaction (PCR). They concluded that an instrument would be ready for surgery as soon as the print is completed, as long as they were printed on a sterile surface and in a clean environment, such as an operating room [38]. Accordingly, Neches and collaborators observed that the extrusion temperatures used in 3D printing are often significantly higher than the temperatures used in the autoclave cycles. They demonstrated that, from a non-sterile feedstock of thermoplastic like PLA they could produce sterile labware components for a wide variety of applications, without needing any post-fabrication treatment, including applications with bacteria and cell culture. They attributed the bacteria present in some samples to handling errors because they were associated with the microflora of human skin. In any case, they suggested that there could be a difference in where their printer was set (open bench or under a laminar flow hood with or without ultraviolet light) [55]. Aguado-Maestro and co-workers suggested that there is a certain intrinsic sterility in products manufactured by domestic 3D printers. Their results are in line with the previous authors, where the high temperatures and pressure used for filament deposition decreased the bacterial load of the biomodels, going from a high suspension for contamination of the pieces to only a few colonies, obtained after enriched culturing of non-sterilized samples before the 3D printing [15].

Other authors also studied 3D printing as a sterilization method but with ABS as a raw material [56,57]. Kondor and colleagues successfully manufactured a surgical instrument kit where 90% of the parts taken directly after printing were determined to be sterile by biological tests [56]. Skelley and collaborators proposed 3D printing as an intrinsic sterilization process associated with the process of heated extrusion in FDM printing. The sterility of 3D-printed ankle fracture fixation plates and cortical screws (using ABS) were therefore assessed using thioglycollate broth cultures at 24 h, 48 h, and 7 days. The sterility of 100% of all test samples and the bacterial growth in the positive control demonstrated an intrinsic sterilization process suitable for FDM 3D printing in future orthopaedic applications [57]. The “in vitro” environment of this study also limited the ability to generalize the results as being sterile and safe for long term orthopaedic implants.

## 6. Current Status and Future Perspectives

Sterilization is an important and problematic step that should be considered as early as possible in the design of any new medical device intended to be use in contact with sterile tissues, mucous membranes, or breached skin, in order to save money, time and trouble [1]. Any failure associated with the sterilization could trigger significant institutional costs related to disease transmission in a bad reuse or with the appearance of nosocomial infections in patients. Thanks to advances in device sterilization methods, fortunately, most nosocomial infections today are not related to this issue. However, it is important to consider sterilization issues and requirements at the earliest stages of the development of any new medical device, to ensure that the final product can be sterilized in an effective and safe manner, with the most cost-effective and environmentally friendly procedures [1]. The vast majority of traditional biomedical devices were designed to withstand traditional sterilization techniques. However, with advances in regenerative medicine and tissue engineering, we can say that we are dealing with a new generation of biomedical biomaterials, much more complex and even patient-specific, thanks to 3D printing. The importance of sterilization or elimination of pathogens has become very relevant nowadays, creating a great social awareness due to the COVID-19 crisis.

Despite this important step after the manufacture of any biomedical device, as we can see in Table 2 and Table 3, only two studies have been found that compared more than two sterilization methods for PLA, analysing both physicochemical and biocompatibility changes [23,35]. Since PLA is a thermal and hydrolytic sensitive biomaterial, conventional sterilization techniques such as heat sterilization, gamma irradiation, and ethylene oxide, may not be the ideal methods for sterilization of PLA. It can be concluded that saturated steam heat (autoclave) is discarded by most authors causing complete deformations and profound structural changes. Although gamma irradiation causes molecular weight changes and chain scissions among others, these changes were considered by most authors as not significant and not affecting biocompatibility, thus accepting this method in many cases. Ethylene oxide also produced some changes in PLA, but although its major problem is toxicity and residues, some authors demonstrated the biocompatibility of the materials after sterilization with this method. Other techniques such as E-beam and HPGP have also been described to sterilize PLA effectively without producing severe changes. E-beam produces less degradation than gamma irradiation, but the penetrating power is dependent on the kinetic energy and the density of the biomaterial, causing more damage as the energy increases. The HPGP sterilization seems to be a promising technique for many authors. However, some authors do not recommend gas plasma as a sterilization method for surgical guides and PLA biomodels with voids as complete sterilization was not achieved. Its lower penetration depth compared to other techniques may be the reason why it is most used as a surface sterilization technique for implantable devices and polymers.

The new scCO_2_ sterilization technique is strongly emerging as an effective technique for the sterilization of sensitive materials. Despite the fact that this technique has taken its first steps in some regulatory agencies, it is necessary to study this technique in detail, to guarantee a correct sterilization and preservation of the bio functionality of the materials, at the same time. Parameters such as pressure, temperature, time, and the use of additives are key for a regulation of the methodology and standardization.

Recently, some authors were also discussing the 3D printing process as a self-sterilization technique, although all biomodels or scaffolds still need to undergo a sterilization process before being used in an operating room to avoid risks of contamination during the manufacturing process and especially if the working environment is not completely sterile. In the specific case of 3D-printed devices, special care must be taken because they are usually hollow. As detailed by Aguado-Maestro [15], it is very important that sterilization is also effective inside the voids in case that a model breaks during a surgical intervention. This is the first publication regarding the sterilization methods of in-hospital manufactured 3D-printed biomodels in polylactic acid, but a more in-depth study would be needed in which physical-chemical and biological changes are analysed with more techniques and not only in terms of deformation and sterilization.

Although promising new techniques such as supercritical CO_2_ have emerged, much needs to be done to expand knowledge regarding sterilization methods for sensitive materials. Research related to new sterilization methods is also required, that address many of the limitations of current techniques, where many single-use biomedical devices can be reused, with clear economic benefits. The effectiveness and post-sterilization effects of new emerging techniques need therefore to be further investigated before they can be declared safe and effective for their use.

**Table 2:** Summary of the state-of-the-art since 1990 regarding the sterilization of polylactic acid (PLA) based materials for biomedical devices.

Material	Sterilization Method	Characterization Method		Changes after Sterilization	References
		Physicochemical Evaluation	Biological Evaluation		
PLA	Steam heat	Molecular weight Mechanical properties	-	Yes	[22]
Lactide copolymers	Dry heat	Molecular weight/Mechanical properties/DSC	-	Yes	[21]
PLLA	EtO Gamma radiation	Molecular weight (GPC)/Mechanical tests/FTIR/DSC/Degradation studies	-	No Yes	[33]
Microspheres of PLA and PLGA	scCO_2_	Degradation analysis/DSC/FTIR	Microbiological	No	[53]
Poly (96 L/4D-lactide)	Steam heat EtO Gamma irradiation	Mass loss/Molecular weight/DSC/Degradation studies	Cytotoxicity	Yes Yes Yes	[23]
Spin-cast films Me.PEG-PLA copolymer	UV radiation	Protein adsorption (XPS)/Surface topography (AFM)/Molecular weight (GPC)/Composition (H-NMR)/Water soluble fraction (GPC)	Cell adhesion	No (in 2 h) Yes (after 5 to 24 h)	[51]
PLLA pellets	EtO	Mechanical properties/Molecular weight/DSC/GPC/XRD/Raman	-	Yes (slight changes)	[34]
PLA orthopaedic implant	HPGP (Sterrad) EtO	Molecular weight (GPC)/DSC/Mechanical properties/WAXD/Contact angle/ATR-FTIR/H2O2 residuals	-	Yes Yes	[36]
Fluconazole- PLA or PLLA implantable delivery rods	Gamma radiation	Loading efficiency/PLC/XRD/GPC	In vivo release assays	Yes	[44]
Hydroxyapatite/PLLA composite biomaterial	Gamma radiation	SEM/GPC/TGA/Mechanical properties	-	Yes (acceptable)	[10]
PLA films	UV radiation	Molecular weight (GPC)/Contact angle	-	Yes	[52]
PLA ultrasound contrast agents	O_2_ Plasma	Acoustic properties/Surface morphology/Zeta potential	-	Yes	[41]
Poly-L-lactide electrospun scaffold	Absolute ethanol Dry oven Steam heat UV radiation HPGP	SEM/ATR-FTIR/DSC	Microbiological sterility assay	Yes (UV and HPGP the most efficient)	[28]
PLA based ultrathin fibers for osteoconductive bone scaffolds	Gamma radiation	SEM/ATR-FTIR/DSC/TGA	Cell viability Cell anchorage	No	[45]
3D scaffolds and 2D film with a graft copolymer of PLA for tissue engineering	Gamma radiation	-	Cytotoxicity	Yes	[42]
PLA and PLGA guided tissue regeneration	Gamma radiation EtO (only PLAG)	FTIR/DSC/TGA/SEM	Microbiological	Yes	[46]
PLA (70:30) coated with plasma polymerized Allylamine fibre meshes	Gamma radiation	XPS	Cell morphology In vivo studies	No and changes in cell spreading	[47]
PLLA porous scaffolds	scCO_2_	DSC/SEM/Crystallinity	Microbiological Biocompatibility	No	[54]
Electrospun PLA fiber alignment for biomedical applications	EtO UV irradiation Gamma irradiation	FTIR/DSC/Contact angle/SEM/Fibre alignment quantification (FFT)	Cell adhesion Cell proliferation	Yes No No	[35]
PLA films	Saturated steam Ethylene oxide HPGP E-beam radiation Gamma radiation	ATR-FTIR/DSC/Contact angle/Crystallinity/Colorimetry	-	Yes (not recommended) The rest of techniques do not produce significant changes	[26]
PLA	Low temperature plasma	-	Mortality of several microorganisms	-	[40]
PLA films	EtO	TGA/DSC/FTIR	Citotoxicity (MTT) in vivo Histology	No	[37]
PLA flat sheets (for single-use, disposable medical devices)	Saturated steam EtO E-beam HPGP	Molecular weight (GPC)/WAXD/DSC/FTIR/Mechanical properties	-	EtO and saturated steam are discarded. Recommends the use of e-beam and HPGP	[27]
PLA thin films for corneal implants	Steam sterilization	SEM/Contact angle/ Surface topography	In vivo assays (implants in corneal rabbits)	Yes	[25]
PLA thin films	Steam sterilization	SEM/Surface topography/Contact angle/FTIR		Yes	[24]
Commercial PLA	E-beam	Molecular weight/Yellow index/WAXD/DSC/Mechanical properties	-	Yes (at higher doses)	[48]
PLA films	E-beam Gamma radiation	Color analysis/surface tension/FTIR/DSC/Mechanical properties/Molecular weight/Permeability	-	Yes	[50]

Abbreviation: EtO: ethylene oxide; HPHP: hydrogen peroxide gas plasma; UV: ultraviolet; scCO_2_: supercritical carbon dioxide; e-beam: electron-beam.

**Table 3:** Summary of the published papers related to the sterilization of 3D printing of PLA for biomedical devices.

3D Printing Materials	Sterilization Method	Characterization Method		Effects of Sterilization	References
		Physicochemical Evaluation	Biological Evaluation		
PLA for biomodels	Autoclave	Changes in area, volume and deformity by scanning	Sterility tests	Following their printing protocols and autoclave at 134 °C the pieces, it is safe and does not significantly alter the morphology of biomodels	[31]
HTPLA custom cutting guides (CCG) for pediatric orthopaedic surgery	Autoclave	Design geometry (visual inspection) Mechanical properties	-	A HTPLA-printed CCG was produced and sterilized aggressively, maintaining its mechanical properties and design geometry	[32]
PLA pieces	Autoclave	Mechanical resistance (breaking load/deformation/permeability)	Sterility tests	Autoclave sterilization of PLA-printed pieces is safe for the patient and mechanically strong for the surgeon	[30]
PLA cylinders for bone model	EtO Gas Plasma Steam heat (autoclave)	Visual deformation	Bacterial growth of contaminated cylinders	Steam heat deformed completely the pieces. Gas Plasma does not eliminate all microorganisms Recommends the EtO in hospitals	[15]
PLA Genioplasty Guide	HPGP (Sterrad^®^)	Volumetric deformation	-	Acceptable for surgical use (<1 mm)	[39]
PLA bone model	Autoclave	Size analysis	-	Acceptable for surgical use (<1 mm)	[29]
PLA scaffolds	Gamma radiation	-	Cytotoxicity Live/Dead assay	Biocompatible scaffolds, with bone cell colonization	[43]
PLA for laboratory equipment	3D printing extrusion	-	Cell culture Microbiological tests	The extrusion process sterilizes the piece, with possible applications in experiments with bacteria and cells	[55]
PLA (1% gentamicin) for biomedical applications	H_2_O_2_ vapour	Mechanical properties Physical changes (dimension, mass, colour)	-	Changes in colour, mass and mechanical properties, which may not be significant depending on the application	[11]
PLA surgical retractors	2.4% glutaraldehyde solution 3D printing extrusion	Strength test	Polymerase chain reaction (PCR) to test bacterial load	Material extruded in a clean environment produces a ready-to-use sterile instrument. Residuals of bacterial nucleic acids were found after sterilization by glutaraldehyde, but it is attributed to the high sensitivity of the test (residuals belonging to dead bacteria) Strong enough for the demands of the operating room.	[38]

Abbreviation: EtO: ethylene oxide; HPGP: hydrogen peroxide gas plasma.

## 7. Conclusions

As indicated in this review, PLA is a widely used biomaterial for the processing of implantable devices, scaffolds, instruments, guides, or models. Since these devices will be in direct contact with the human body, it is a critical task to choose the appropriate technique to effectively sterilize but at the same time to maintain structural and physicochemical integrity, without compromising the biocompatibility.

This paper aims to help researchers to choose the best sterilization method and better understand the changes related to PLA. It is clear, that no suitable “perfect sterilization technique” for PLA exists and the choice should be based on the type of product to be sterilised and the applications for which it will be manufactured as well as the best available techniques in each case and each moment. A thorough analysis of the changes should always be made to avoid compromising their function. Moreover, the operation conditions of a chosen sterilization technique should be precisely controlled and evaluated case by case.

It can be concluded that autoclave is discarded by most authors, except in 3D printing, which seems to be gaining relevance. Ionising radiation (gamma radiation and electron beam) can be effective as long as the dose is controlled to avoid severe changes. The HPGP and new scCO_2_ seem to be promising sterilization techniques. In addition, more studies are needed to evaluate the changes produced by the sterilization process especially in novel and sensitive biomaterials. As for 3D printing, this technique must also be provided with the necessary protocols and validation so that its use in hospitals can be applied easily, safely, comfortably, and universally.

## Figures and Tables

**Table 1:** Comparison between currently used sterilization techniques [5,6,8].

Method	Technique	Advantages	Disadvantages
Heat	Dry heat/steam	Nontoxic residues, low cost, simple, fast, effective, good penetration	Not suitable for heat-and/or moisture-sensitive materials like biodegradable polymers
Chemical	Ethylene oxide	Low-temperature setting for heat-and/or moisture-sensitive materials, effective, good penetration	Potential hazards to staff and patients Toxic, flammable, and carcinogenic Long treatment/aeration time needed
	Peracetic acid	Low temperature, no activation required, odour or irritation not significant	Materials compatibility concerns, limited clinical use (only for immersible instruments/materials), no long-term sterile storage possible
Irradiation	Gamma irradiation	Nontoxic residues, low temperature, good penetration	Damaging polymers and biological materials High cost
	E-beam	Nontoxic residues, low temperature, short treatment time	Damaging polymers and biological materials, limited penetration distance
Plasma	H_2_O_2_ gas plasma	Nontoxic residues, low temperature setting suitable for heat-and/or moisture-sensitive materials	Not suitable for cellulose (paper), linens and liquids, and devices with hollows May cause changes in chemical and mechanical properties of polymers, produce reactive residuals
